# Diffuse Large B-cell Lymphoma in a Young Patient Presenting as a Cecal Mass

**DOI:** 10.7759/cureus.31632

**Published:** 2022-11-18

**Authors:** Haider Ghazanfar, Abhilasha Jyala, Haozhe Sun, Elona Shehi, Muhammad Sulh, Harish Patel

**Affiliations:** 1 Internal Medicine, BronxCare Health System, Bronx, USA; 2 Medicine/Internal Medicine, BronxCare Health System, Bronx, USA; 3 Medicine/Gastroenterology, BronxCare Health System, Bronx, USA; 4 Pathology, BronxCare Health System, Icahn School of Medicine at Mount Sinai, Bronx, USA

**Keywords:** diffuse large b-cell lymphoma, cecal mass, colonoscopy, malignancy, abdominal mass, lymphoma, cecum

## Abstract

Colorectal lymphomas are very rare and are usually found in male patients between the fifth and seventh decade of life. Our patient was a 30-year-old male who presented with the complaint of epigastric pain and abdominal mass for the past three months. Physical examination was remarkable for soft solid abdominal mass extending from the right side of the abdomen toward the left side. Computed tomography showed a large lobulated mass in the right lower quadrant involving multiple loops of bowel. The mass measured 15.1 x 12.5 cm in transverse dimension and 16.2 cm in craniocaudal dimension. Colonoscopy showed a villous and fungating mass occupying the whole cecum and extending into the ascending colon. Pathology from the colonoscopy mass revealed diffuse large B-cell lymphoma, which was CD20 (cluster of differentiation 20) positive. He was started on rituximab, cyclophosphamide, doxorubicin, vincristine, and prednisone (R-CHOP) chemotherapy regimen and is following at the oncology outpatient department. A combination of surgical resection and chemotherapy is used in treating patients with colorectal lymphoma.

## Introduction

Lymphomas are categorized into two groups: non-Hodgkin's lymphoma and Hodgkin's lymphoma. Non-Hodgkin's lymphoma is the seventh most common type of cancer in the United States, and as of August 30, 2022, accounted for 4.2% of all new cancer cases diagnosed in the US in 2022 [1]. The gastrointestinal tract accounts for 30-40% of extranodal non-Hodgkin's lymphoma [2]. Primary gastrointestinal lymphomas are rare and account for only 1-4% of all gastrointestinal malignancies and 10-15% of all non-Hodgkin's lymphoma [3,4]. Approximately 60-75% of the cases occur in the stomach [4,5]. Primary lymphoma of the colon accounts for only 0.2-0.6% of all colon cancer [6]. Primary colorectal lymphoma is more common between the fifth and seventh decade of life and is more common in males as compared to females [7,8]. Abdominal pain, weight loss, change in bowel movement, and abdominal mass are the most common symptoms of colorectal lymphoma. Intestinal obstruction due to colorectal lymphoma is rare due to its pliable nature. Fever and drenching night sweats are usually not seen in patients with colorectal lymphoma. Cecum was found to be the most frequent site of colorectal lymphoma in 45-60% of the patients [9,10]. This might be due to the presence of a large amount of lymphoid tissue in that area as compared to the rest of the colon [11]. We present a case of primary cecal lymphoma in a 30-year-old male who presented with abdominal mass and epigastric pain.

## Case presentation

Our patient was a 30-year-old Hispanic male who presented to the emergency department with a complaint of epigastric pain and abdominal mass for the past three months. His epigastric pain was sharp in character, non-radiating, mild in intensity, was not associated with eating or fasting, and had progressively increased with time. His abdominal mass had progressively increased with time and he was unable to lie in a prone position. He also reported nausea and early satiety. He stated that he has lost 30 pounds over three months. He stated that the main reason he was concerned now was because of the inability to lie on his bed in the prone position. He denied dysphagia, vomiting, hematemesis, diarrhea, constipation, change in stool consistency and caliber, hematochezia, and melena.

He denied any significant past medical and surgical history. His family history was significant for stomach cancer in two of his maternal aunt. He denied smoking and using any recreational drugs.

In the emergency department, he was found to have a temperature of 98.4°F, heart rate of 84 beats per minute, respiratory rate of 16 breaths per minute, blood pressure of 121/66 mmHg, and was saturating 100% on room air. Abdominal examination was significant for a soft solid mass palpated, which was extending from the right side of the abdomen toward the left side. There was no abdominal tenderness or rigidity. He was found to have normal bowel sounds. He had no cervical and inguinal lymphadenopathy. He had bilateral vesicular breathing on lung auscultation and had normal heart sounds. Neurological examination was unremarkable. He had no cervical, axillary, or inguinal lymphadenopathy. His initial laboratory findings have been presented in Table 1.

**Table 1:** Laboratory values at the time of admission

Laboratory parameter	Value	Reference range
White blood cell count	7.6	4.8-10.8 k/ul
Hemoglobin	9.2	12.0-16.0 g/dl
Hematocrit	30.9	42.0-51.0%
Mean corpuscular volume	64.8	80.0-96.0 fL
Platelet	368	150-400 k/ul
Sodium	139	135-145 mEq/L
Potassium	4.5	3.5-5.0 mEq/L
Bicarbonate	27	24-30 mEq/L
Chloride	101	98-108 mEq/L
Glucose	77	70-120 mg/dL
Blood urea nitrogen	11	8.0-26.0 mg/dL
Creatinine	0.6	0.5-1.5 mg/dL
Calcium	8.8	8.5-10.5 mg/dL
Albumin	4	3.4-4.8 g/dl
Total bilirubin	0.3	0.2-1.2 mg/dL
Direct bilirubin	<0.2	0.0-0.3 mg/dL
Alkaline phosphatase	56	53-128 unit/L
Aspartate transaminase	13	9-48 unit/L
Alanine aminotransferase	13	5-40 unit/L
Total protein	6.6	6.0-8.5 g/dl

**Figure 1:**
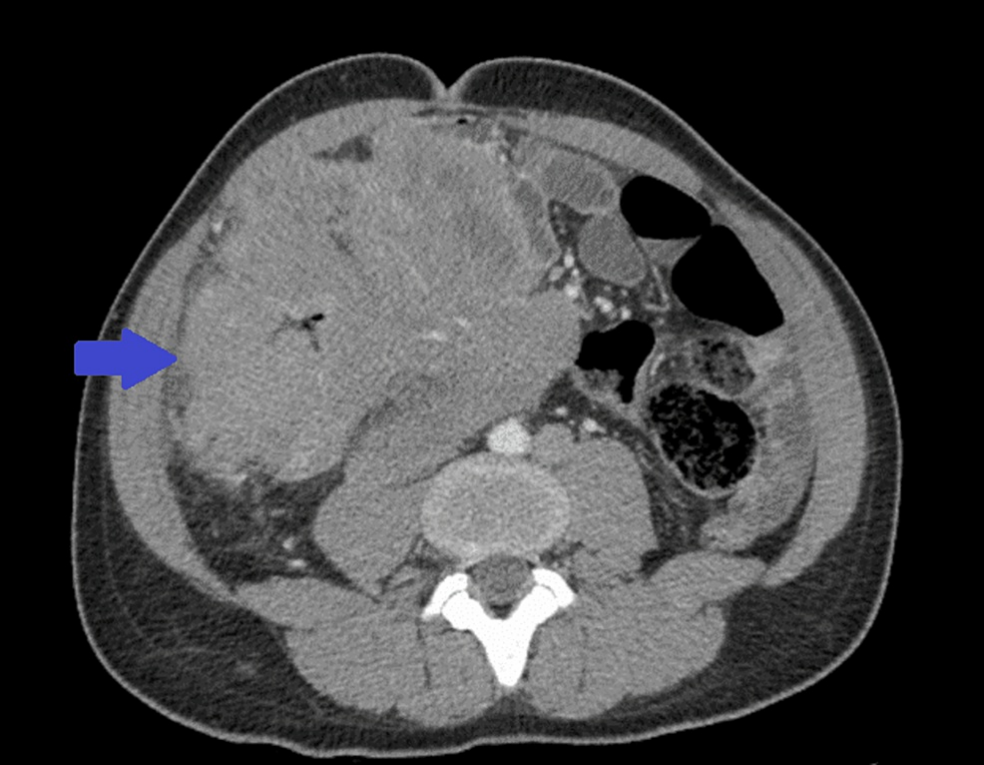
Computed tomography of the abdomen and pelvis with intravenous contrast showing a large lobulated mass in the right lower quadrant involving multiple loops of the bowel (blue arrow)

The patient underwent computed tomography (CT) of the abdomen and pelvis with intravenous contrast, which showed a large lobulated mass in the right lower quadrant involving multiple loops of bowel. The mass measured 15.1 x 12.5 cm in transverse dimension and 16.2 cm in craniocaudal dimension. This has been shown in Figure 1.

**Figure 2:**
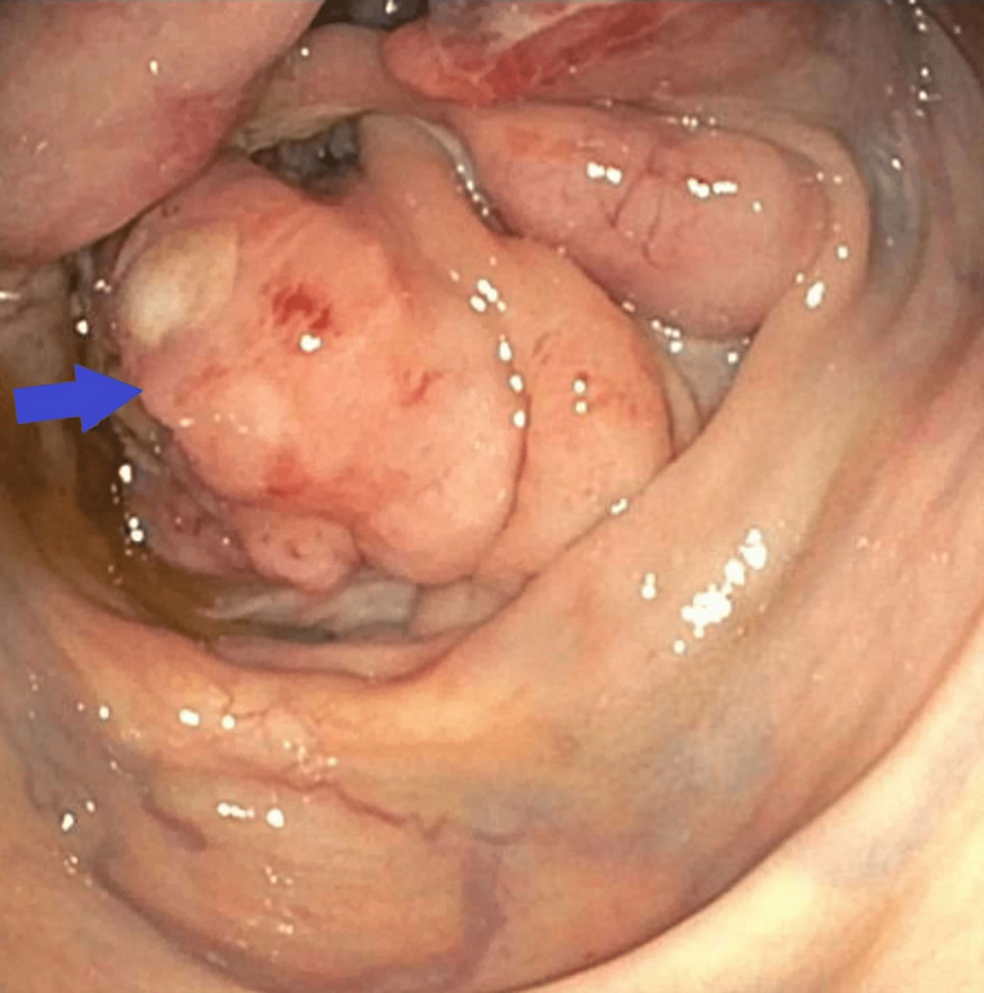
Fungating and friable mass occupying the cecum and extending into the ascending colon (blue arrow)

He was given pain medication and antiemetics in the emergency department. He was admitted to the floor for further workup. Colonoscopy showed a villous and fungating mass occupying the whole cecum and extending into the ascending colon. The mass was biopsied and sent for analysis. The appendix and ileocecal valve could not be identified. This has been shown in Figure 2.

He underwent esophagogastroduodenoscopy (EGD) for his epigastric pain, which was significant for erythema of the greater curvature of the gastric body. His cancer antigen 19-9 (CA 19-9) and carcinoembryonic antigen (CEA) were unremarkable. His peripheral smear was unremarkable. His sputum acid-fast bacilli (AFB) culture was negative. He tested negative for human immunodeficiency virus (HIV). He underwent a CT of the chest and head, which was unremarkable.

**Figure 3:**
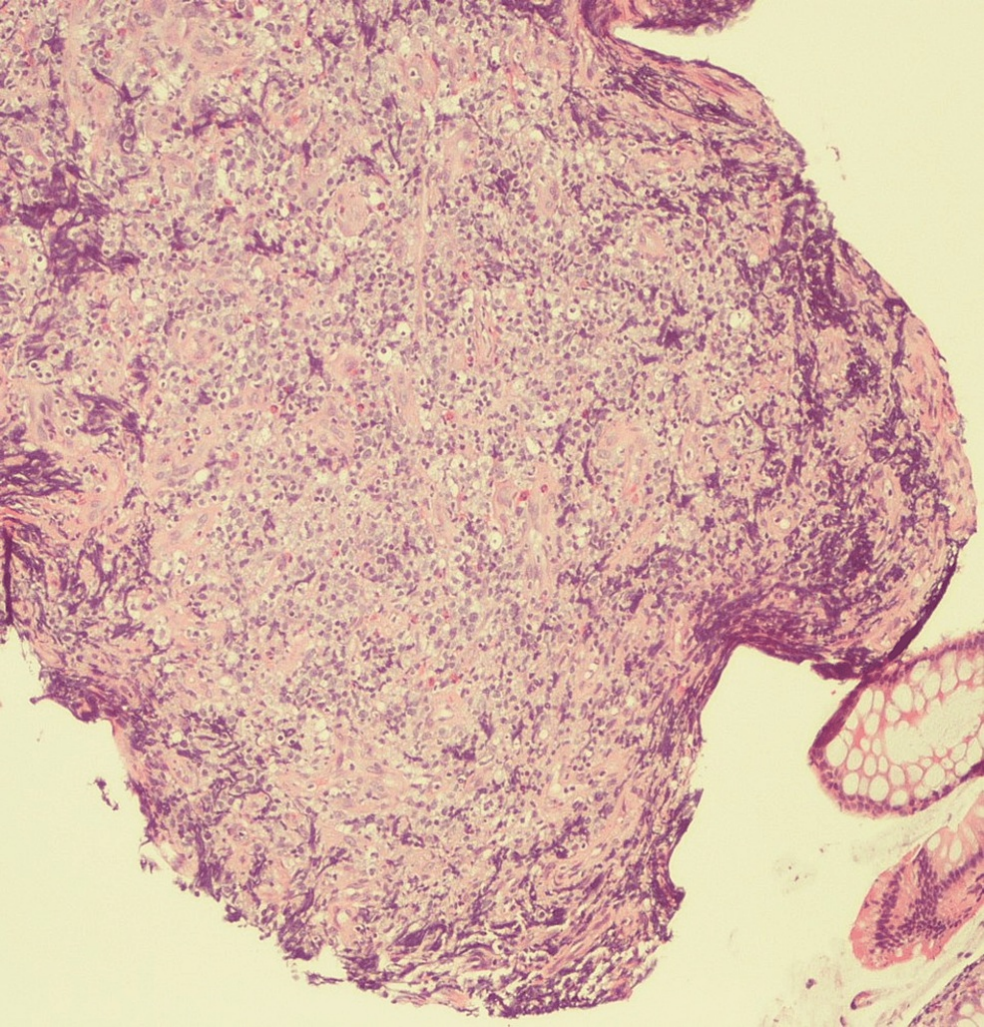
Low-power hematoxylin and eosin stain showing sheets of large pleomorphic lymphocytes

**Figure 4:**
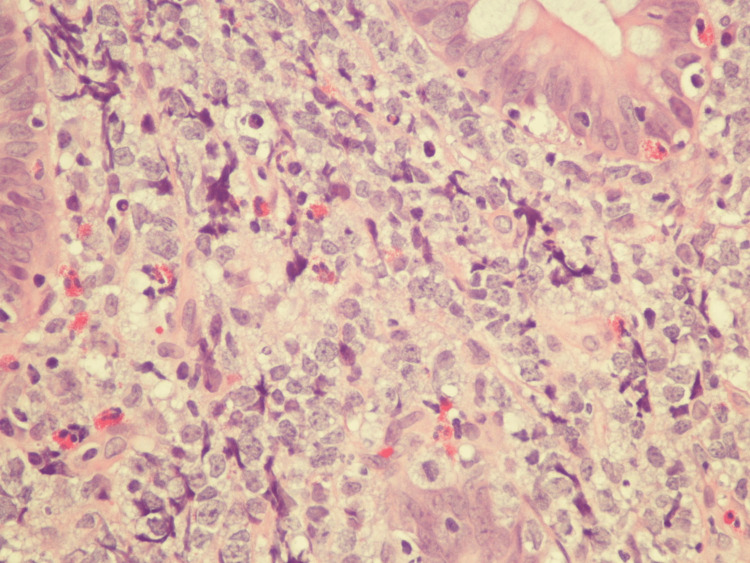
High-power hematoxylin and eosin stain showing fragments of colonic mucosa with sheets of large pleomorphic lymphocytes with irregular nuclei, vesicular chromatin, variably prominent nucleoli, and a moderate amount of cytoplasm

Pathology from the colonoscopy mass revealed diffuse large B-cell lymphoma, which was CD20 (cluster of differentiation 20) positive. Ki-67 showed a high proliferative index (-80%) and Epstein-Barr virus-encoded RNA-1 in situ hybridization (EBER(ISH)) stain was negative. This has been presented in Figures 3, 4.

Biopsy from the greater curvature of the gastric body showed mild gastritis with focal activity and no *Helicobacter pylori* were seen. He was diagnosed with diffuse large B-cell cecal lymphoma. He was started on rituximab, cyclophosphamide, doxorubicin, vincristine, and prednisone (R-CHOP) chemotherapeutic regimen. He underwent two cycles of chemotherapy and had a significant decrease in abdominal mass size on physical examination. The patient is currently followed in the oncology clinic for repeat CT scans, positron emission tomography (PET), and chemotherapy.

## Discussion

*H. pylori* infection, autoimmune diseases, immunodeficiency, immunosuppression, celiac disease, and inflammatory bowel disease are a few conditions that increase the risk of getting gastrointestinal lymphoma. *H. pylori* infection has been associated with the development of mucosa-associated lymphoid tissue (MALT) lymphoma of the stomach [12]. Currently, the role of *H. pylori* infection in the development of colon lymphoma has not been established [13]. Our patient did not have any *H. pylori* infection and he tested negative for HIV.

Differential diagnoses of large cecal masses in a young patient include adenocarcinoma, lymphoma, gastrointestinal stromal tumors, and tuberculosis. Patients with large cecal masses usually present with the sign of obstruction but our patient was able to tolerate food and had no clinical sign of obstruction despite the mass being 15.1 x 12.5 x 16.2 cm in size.

CT of the abdomen and double contrast barium enema are the two major radiological modalities in diagnosing colon lymphoma. Radiological findings of colorectal lymphoma on CT scan include polypoid mass, circumferential infiltrative lesion, large cavitary lesion, or ulcerative lesion [14,15]. Polypoid masses are the most frequent radiological finding in patients with primary colorectal lymphoma and they usually are located near the ileocecal valve region [14]. Polypoid colorectal lymphoma is usually larger as compared to colon adenocarcinoma and can extend up to 20 cm in size. Our patient was found to have a large polypoid mass extending up to 16.2 cm in its longest dimension on the CT scan. A colonoscopy with a biopsy of the mass is the gold standard for confirming the diagnosis of colorectal lymphoma. Primary colorectal lymphoma is classified into three major subtypes based on colonic features. According to a retrospective chart review, the ulcerative type was seen in 64% of patients, the polypoid type was seen in 16% of patients, and the massive type was seen in 20% of patients [16]. A retrospective study done in Spain covering over 23 years reported that diffuse infiltration was seen in 41.7% of patients while mass was seen in 33.7% of patients [17]. Large B-cell lymphoma is the most common histologic subtype of non-Hodgkin’s lymphoma found in patients with colonic lymphoma [9].

A combination of surgical resection and chemotherapy is used in treating patients with colorectal lymphoma. The CHOP (cyclophosphamide, hydroxydaunorubicin, oncovin, and prednisone) chemotherapeutic regimen is the treatment of choice in patients with colorectal lymphoma. Our patient had a good response to chemotherapy so surgical intervention was not considered. The addition of rituximab to the CHOP chemotherapeutic regimen has been shown to result in a higher response rate and also improves the progression-free, event-free, disease-free, and overall survival rates [7,18]. The stage and histological grade of colorectal lymphoma are the most important prognostic factors in these patients [7,9]. A review of the literature on patients diagnosed with cecal lymphoma has been summarized in Table 2 [18-21].

**Table 2:** Review of the literature on cecal lymphoma

Author	Age	Gender	Clinical signs and symptom	HIV status	CT scan finding	Type of lymphoma
Sangma et al. [18]	27	Female	Abdominal pain, nausea, and vomiting	Unknown	Lobulated thick wall lesion in the ileocecal, cecum, and colon	Burkitt lymphoma
Yehya et al. [19]	44	Male	Severe abdominal pain	Positive	Diffuse cecal wall thickening	Diffuse large B-cell lymphoma
Jayabackthan et al. [20]	35	Female	Abdominal pain	Unknown	Ileocolic intussusception with a mass lesion	Mucosa-associated lymphoid tissue lymphoma
Kudaş et al. [21]	20	Female	Abdominal pain, nausea, and vomiting	Unknown	Cecal mass	Diffuse large B-cell lymphoma

Based on our literature review of published cases of cecal lymphoma in patients older than 18 years of age but less than 45 years of age, in most of the case reports, the HIV status was unknown or positive. Our patient did not have vomiting and was HIV-negative.

## Conclusions

Primary colonic lymphoma is a very rare malignancy and is most commonly seen in elderly male patients. Our case highlights the importance of having awareness of colonic lymphoma in young patients who do not have risk factors predisposing to colonic lymphoma. Early diagnosis and prompt treatment are fundamental in improving the overall prognosis for the patient.
